# The Reactive Species Interactome in Red Blood Cells: Oxidants, Antioxidants, and Molecular Targets

**DOI:** 10.3390/antiox12091736

**Published:** 2023-09-07

**Authors:** Miriam M. Cortese-Krott

**Affiliations:** 1 Myocardial Infarction Research Laboratory, Department of Cardiology, Pulmonology and Angiology, Medical Faculty, Heinrich-Heine-University, Universitätstrasse 1, 40225 Düsseldorf, Germany; miriam.cortese@hhu.de; 2 Department of Physiology and Pharmacology, Karolinska Institutet, 17177 Stockholm, Sweden; 3 CARID, Cardiovascular Research Institute, Heinrich-Heine University, 40225 Düsseldorf, Germany

**Keywords:** red blood cells, reactive species interactome, nitric oxide, sulfide, antioxidant enzymes, oxidative stress, hemoglobin

## Abstract

Beyond their established role as oxygen carriers, red blood cells have recently been found to contribute to systemic NO and sulfide metabolism and act as potent circulating antioxidant cells. Emerging evidence indicates that reactive species derived from the metabolism of O_2_, NO, and H_2_S can interact with each other, potentially influencing common biological targets. These interactions have been encompassed in the concept of the *reactive species interactome.* This review explores the potential application of the concept of *reactive species interactome* to understand the redox physiology of RBCs. It specifically examines how *reactive species* are generated and detoxified, their interactions with each other, and their targets. Hemoglobin is a key player in the *reactive species interactome* within RBCs, given its abundance and fundamental role in O_2_/CO_2_ exchange, NO transport/metabolism, and sulfur species binding/production. Future research should focus on understanding how modulation of the *reactive species interactome* may regulate RBC biology, physiology, and their systemic effects.

## 1. Introduction

Oxidative stress was originally defined by Helmut Sies as “*an imbalance between antioxidants and oxidants in favor of the oxidant, which can potentially lead to molecular damage*” [1]. Excessive production of oxidants and/or consumption of antioxidant systems disrupt cellular redox control and signaling [2,3]. He also introduced the term “reactive oxygen species” (ROS) to describe oxidants derived from the metabolic utilization of oxygen, including free oxygen radicals (^•^OH), superoxide radical anion (^•^O_2_^−^), and hydrogen peroxide (H_2_O_2_).

Intracellular antioxidant systems responsible for scavenging and removal of ROS comprise molecules that act as redox couples and/or cofactors of enzymes (including the “*inevitable*” glutathione [4], as well as antioxidant enzymes, like glutathione peroxidase, catalase, and superoxide dismutase. The concept of cellular redox balance/disbalance was described by using the image of a traditional scale consisting of two plates suspended at equal distances from a fulcrum. Antioxidants were depicted on one plate, and oxidants (or ROS) were depicted on the other plate. In a weighing balance, the weighing plates level off only when a static equilibrium between the two plates is achieved, i.e., only when the masses on the two plates are equal.

According to this conceptual model, under resting conditions, the cellular redox state is maintained when the oxidant production is balanced by the intracellular antioxidant response. If the levels of oxidants exceed those of antioxidants, as a result of higher ROS levels or lower antioxidant production [1], there will be damage to the cell. ROS have been shown to react with other endogenously produced free radicals and reactive species containing nitrogen (reactive nitrogen species, RNS) and sulfur (reactive sulfur species, RSN), which have also been proven to be potent oxidants and disturb normal redox homeostasis [5]. In earlier studies, the presence of enhanced oxidative stress or ROS/RNS/RSS levels was associated with diseases such as diabetes, inflammation, and mitochondrial dysfunction [1].

The study of redox biology and its conceptualization has evolved considerably since then. It was proven that ROS are not the only reactive species responsible for oxidative damage. The free radical nitric oxide (NO) was demonstrated to be produced endogenously in cells by a nitric oxide synthase (NOS) and act as a signaling molecule and a neurotransmitter. Also, H_2_O_2_ was found to have important signaling functions under physiological conditions and be produced under highly controlled conditions by NADPH oxidases. In recent years, sulfide has been shown to be produced endogenously in mammalian cells and tissues and shows fundamental biological and physiological effects, resembling the effects of NO in some cases. Here, the term “sulfide” is used for simplicity to indicate the mixture of all sulfide species found in equilibrium among themselves at biological pH., i.e., H_2_S (g), H_2_S (aq), HS^−^, and S^2−^. In water at pH 7.4, H_2_S (aq) is rapidly equilibrated into HS^−^ (>70%) and S^2−^. Therefore, the main biologically relevant sulfide species in cells are H_2_S (aq) and HS^−^ (>70%). The IUPAC nomenclature of these species is as follows: H_2_S, sulfane or hydrogen sulfide; HS^−^, sulfanide or hydrogen(sulfide)(-1); S^2−^, sulfide(-2) or sulfanediide.

It is also clear that these radicals and oxidants are highly compartmentalized, undergo chemical reactions and complex interactions among them, and may affect common biological targets. In other words, in a biological environment, molecules derived from the metabolism of O_2,_ NO, and H_2_S may interact with each other and should be considered components of the same system. Therefore, we introduced the term *reactive species interactome* [5]. The *reactive species interactome* is defined as an “oxidation-reduction system consisting of chemical interactions between reactive sulfur species (RSS), reactive nitrogen species (RNS), and ROS with their thiol targets”.

Red blood cells (RBCs) are simple, short-lived, anucleated cells present in almost all vertebrates, whose main function is the transport of oxygen (O_2_) and carbon dioxide (CO_2_) along the vascular system of vertebrates. For many years, scientists have thought that the sole function of RBCs was to deliver O_2_ to body tissues. However, there is growing evidence that these cells may be involved in complex systemic redox regulation [6]. RBCs have a very complex and poorly understood *reactive species interactome*.

The presence of high concentrations of hemoglobin and its oxidized form, methemoglobin, is one of the main sources of ROS in RBCs. RBCs are particularly rich in antioxidants and detoxifying enzymes and transport high millimolar concentrations of glutathione [6,7] and glutathione persulfide (GS(S)_n_SG) [8].

In addition, RBCs are exposed to endothelial-derived nitric oxide (NO) and its metabolites, such as nitrite (NO_2_^−^), nitrate (NO_3_^−^), and nitrosospecies (RXNO) [9]. (RXNO indicates low-molecular-weight molecules where a “nitroso group” is bound to a cysteine (nitrosothiols) or an amino group (nitrosamine) [9].) RBCs have been shown to transport NO metabolites and NO bound to hemoglobin as nitrosylhemoglobin and s-nitrosohemoglobin. RBCs also produce NO under hypoxic conditions and release “NO bioactivity”, i.e., they are able to induce vasodilatation in ex vivo bioassays. In addition, RBCs express an endothelial nitric oxide synthase (eNOS) [10]. Therefore, RBCs play a central role in the systemic regulation of NO [6].

Additionally, RBCs participate in sulfide metabolism. RBCs were shown to scavenge sulfide, transport RSS, and express a 3-mercapto sulfotransferase (3-MST) and thus potentially contribute to the endogenous enzymatic production of sulfide and its metabolites as well [11]. The potential chemical, biochemical, and pharmacological interactions of sulfide with NO in RBCs (or “cross-talk”, as defined in the literature) and their reactions with hemoglobin have also been proposed [5,11,12,13,14,15].

Erythroid cells and reticulocytes (and therefore probably also RBCs) contain high levels of heme oxygenase 1, which catalyzes the degradation of oxidized hemoglobin and thereby also produces carbon monoxide (CO) [16]. The interaction of CO with other reactive species in RBCs (or in other compartments) has not yet been investigated in detail. In general, the role of the *reactive species interactome* in the regulation of RBC physiology and pathophysiology remains unclear. This review summarizes recent evidence on the *reactive species interactome* in RBCs and how it may affect the cardiovascular system. Specifically, we will introduce the concept of the *reactive species interactome* as it applies to RBCs, how oxidants are produced in RBCs and how they are detoxified, the role of RBCs in systemic NO and sulfide metabolism, and their cross-talk.

## 2. Role of RBCs in Systemic Redox Regulation—Oxidant Generation and Antioxidant Systems

RBCs contain complex redox systems that are indispensable for the preservation of cellular integrity, the control of cellular metabolism, and the modulation of cellular shape and flexibility [6]. Predominantly, the generation of ROS in RBCs arises from the autoxidation of oxyhemoglobin. This process transitions the iron from its typical ferrous state (Fe^2+^) to a ferric state (Fe^3+^), resulting in the formation of methemoglobin (metHb) and superoxide anion radical (O_2_^•−^). This shift greatly diminishes the oxygen affinity of the prosthetic group of hemoglobin and is followed by protein degradation. Given the substantial concentration of hemoglobin within RBCs (32 to 36 g/dL), this reaction significantly contributes to ROS formation. However, Hb-Fe^3+^ can be reverted back to Hb-Fe^2+^ via cytochrome b5 reductase, using NADH as an electron donor [6]. Iron can also be released from metHb during its degradation, and—if not scavenged by ferritin [17]—it is a potent generator of ROS, mainly by reactions involving O_2_^•−^ radicals or H_2_O_2_, culminating in the production of highly reactive hydroxyl radicals and hydroxyl anions. Interestingly, both Hb-Fe^2+^ (oxy/deoxyHb) and Hb-Fe^3+^ can react with hydrogen peroxide (H_2_O_2_). This reaction forms ferryl hemoglobin (Hb-Fe^4+^=O), which is a highly oxidizing species that generates secondary radicals and ultimately liberates *free* iron. For a detailed discussion of these reactions, please refer to older but excellent reviews [18,19]. RBCs are also recognized for their ability to produce NO under both normoxic and hypoxic conditions [20]. NO may also react with O_2_^•−^ to produce peroxynitrite, but its formation in RBCs has not been studied.

Hence, it is essential for RBCs to possess robust antioxidant systems, both enzymatic and nonenzymatic, which can neutralize these reactive species and sustain their intracellular levels to a minimum. These systems can be divided into three categories.The first category consists of antioxidant molecules and redox pairs, including reduced/oxidized glutathione (GSH), ascorbate/dehydroascorbate, and α-tocopherol [6]. GSH, a linear tripeptide, can occur in the reduced form (GSH) or in the oxidized form (GSSG, a dimer of two GSH molecules). The ratio of GSH/GSSG in RBCs is utilized to estimate the redox state of an organism; typically, reduced GSH constitutes 90–95% of the total GSH [4,5,21,22]. Ascorbate, also referred to as vitamin C, serves as a crucial antioxidant in RBCs. The redox pair ascorbate/dehydroascorbate plays a vital role in sustaining redox homeostasis within RBCs by reducing metHb and oxidants that diffuse into the cell membrane [23]. Vitamin E, or α-tocopherol, is another significant antioxidant within RBCs. It is found within the membranes of RBCs due to its lipophilic properties [24].The second category consists of redox equivalents, such as NADH and NADPH, which play a crucial role by providing reducing equivalents for enzymes catalyzing redox reactions. They are reduced during glycolysis and the pentose phosphate pathway. The deficiency of glucose or malfunction of pentose phosphate pathways can severely impact the membrane integrity and redox homeostasis of RBCs [25,26,27,28].

The third category involves enzymatic antioxidant systems that are crucial for the survival and proper functioning of RBCs [29]. A series of detoxifying enzymes is found in RBCs, including catalase, peroxiredoxin 2, glutathione peroxidase, and glutaredoxin. For instance, catalase and glutathione peroxidase catalyze the conversion of H_2_O_2_ into water [30]. Peroxiredoxin and thioredoxin (which is needed to recycle peroxiredoxin) detoxify mainly lipid peroxides in the membrane [31], and play a fundamental role in RBC redox homeostasis in vivo [32]. Glutaredoxins are responsible for keeping thiols in a reduced state; they were isolated from RBCs almost 30 years ago, but their role is still elusive [33,34].

The key function of the enzymatic and nonenzymatic redox antioxidant systems found in RBCs is to keep hemoglobin in a reduced form, thereby preserving its ability to bind oxygen. By limiting the generation of metHb, these systems reduce ROS generation and protect the cellular membrane lipids, proteins, channels, and metabolic enzymes from oxidative stress.

Furthermore, an additional fourth category of multifunctional or “moonlighting” enzymes participate in the overall redox homeostasis by coordinating and fine-tuning antioxidant response, iron homeostasis, and energy metabolism. In RBCs, these include the glyceraldehyde 3-phosphate dehydrogenase (GAPDH), which forms a membrane “metabolome” complex at the N-terminus of Band3/anion exchange 1 (AE-1) together with glycolytic enzymes and cytoskeletal proteins, and can migrate into the cytoplasmic compartment in dependency of the pO_2_ [35,36]. Under oxidative stress conditions and during RBC storage (for example, in transfusion units), the oxidation of Cys152 and His179 in the catalytic pocket of GAPDH alters its metabolic function and leads to a metabolic switching from glycolysis towards the pentose phosphate pathway [37].

Deficiencies in these antioxidant systems can lead to cellular damage and dysfunction derived from the oxidation of redox switches found in cytoskeletal proteins (like spectrin, ankyrin, protein 4.2, and actin), leading to a loss of membrane integrity, altered RBC deformability, and hemolysis [38]. Genetic mutations of these proteins are linked with severe conditions like hemolytic anemia [6]. Moreover, these modifications also occur during RBC storage and are defined as a “storage lesion” [26]. Very elegant proteomics and metabolomics studies carried out in recent years have revealed how pO_2_, protein–protein interactions, redox state, and metabolic control are intimately intertwined and modulate the redox physiology of RBC [37,39]. In addition, these studies may also provide new ways to prevent the “storage lesion” and prolong the therapeutic applicability of transfusion units, which is of fundamental importance in the therapy of different forms of severe anemia and blood loss in the clinic.

## 3. Role of RBCs in Systemic NO Metabolism

RBCs play a pivotal role in regulating the systemic availability and bioactivity of NO by scavenging, binding, and metabolizing NO and its metabolites [20]. In this way, RBCs are an important contributor to the concentrations of other NO metabolites in plasma, including nitrate, nitrite, nitrosothiols (RSNO), and nitrosamines (RNNO). The NO metabolites also show bioactivity as they can release NO (via enzymatic and nonenzymatic reactions) and cause vasodilation, and thereby can affect the cardiovascular system [40,41,42,43,44]. There is also evidence that nitrite may have signaling effects on its own by modulating the activity of downstream targets, including protein kinase A [45,46].

RBCs may regulate the pool of circulating NO metabolites via various mechanisms, mainly involving reactions with hemoglobin or eNOS-derived NO formation [20,47]. Oxyhemoglobin (oxyHb) captures eNOS-derived NO from the vascular endothelium under normoxic conditions in a rapid reaction that leads to the formation of nitrate, thus limiting and fine-tuning the bioactivity of endothelial NO. Under hypoxic conditions, deoxyhemoglobin can also react with NO, forming nitrosyl hemoglobin (HbNO), which is more stable under these conditions. HbNO has been proposed as an indicator of NO bioavailability in RBCs [48]. HbNO was also indicated as being an intermediate for the formation of S-nitroso hemoglobin, which, in turn, initiates a cascade of transnitrosation reactions transferring the nitroso group from one cysteine to the next and mediates hypoxic vasodilation [49,50,51]. In ex vivo experiments, the nitrosation of the membrane protein spectrin aids in preserving RBC deformability under oxidative stress [52]. Deoxyhemoglobin reduces nitrite to NO under hypoxic conditions [53,54,55]. This nitrite reductase activity of deoxyhemoglobin was shown to induce NO-dependent vasodilation under hypoxic conditions, thus offering more evidence that RBCs indeed partake in the vasodilation of vessels and do not merely serve as NO sinks.

RBCs also express an active eNOS, which is, therefore, another source of NO production and is active under normoxic conditions [10]. There is compelling in vivo evidence that endogenous NO production by eNOS in RBCs affects both circulating NO metabolites and blood pressure [56,57]. Chimera mice obtained from the transplantation of bone marrow from eNOS KO into irradiated WT mice showed decreased circulating NO metabolites and elevated systolic blood pressure and mean arterial pressure [56]. Recently, we generated a mouse model lacking eNOS in RBCs obtained by crossing eNOS^flox/flox^ mice with mice expressing a Cre recombinase under the control of the hemoglobin beta chain promoter (HbbCre^pos^ mice) [57]. These mice showed hypertension and increased systemic vascular resistance, accompanied by decreased HbNO levels in RBCs, decreased nitrite/nitrate, and hypertension.

We also generated mice carrying eNOS in RBCs only and lacking eNOS in all other tissues (RBC eNOS KI mice), which we obtained by crossing mice carrying a duplicated and inverted exon 2 (conditional eNOS KO mice) and two pairs of loxP sequences with the HbbCre mice; in the presence of the Cre recombinase, the exon 2 is “flipped”, and eNOS expression is restored. The reactivation of eNOS expression in RBCs rescued the hypertension phenotype and the levels of HbNO in RBCs. These data demonstrate that RBCs play a fundamental role in blood pressure regulation and NO metabolism.

## 4. Role of RBCs in Systemic Sulfide and Persulfide Metabolism

The role of sulfide metabolism in the canonical and noncanonical functions of RBC and/or their dysfunction has never been investigated specifically. However, independent studies have shown that RBCs can scavenge, transport, metabolize, and release sulfide and its metabolites (including thiosulfate, persulfates, and polysulfides) [11]. Therefore, RBCs are likely to play a central role in overall sulfide physiology and pharmacology, similar to their role in NO metabolism.

RBCs rapidly uptake and scavenge high concentrations of sulfide both ex vivo and in vivo [11]. It is very likely that RBCs may contribute in this way to prevent its toxic accumulation in the bloodstream, as proposed already at the beginning of the last century [58,59,60,61].

At physiological pH, sulfide primarily exists as an anion HS^−^, and to a lesser extent, as dissolved H_2_S. In RBCs, sulfide can permeate cell membranes, entering RBCs via the anion exchange protein Band3/AE1, which catalyzes a net acid flux exchanging HCO_3_^−^ [59]. Interestingly, HCO_3_^−^ has a lower affinity for Band3/AE-1 as compared to HS^−^. This explains why, in buffered suspensions, RBCs rapidly take up H_2_S/HS^−^ [59,62]. As a consequence, the uptake of sulfide by RBCs may modulate the pH of the cells and their supernatants. If these effects are also present in vivo, sulfide intake in RBCs may potentially modulate the overall homeostasis of pH in the body.

Under physiological conditions, oxyhemoglobin does not undergo a reaction with sulfide. At very high concentrations, millimolar amounts of sulfide react with oxyhemoglobin to form a stable green compound sulfhemoglobin [63,64,65,66,67]; the reaction requires the presence of oxidative agents and is slower. Intriguingly, sulfhemoglobin is not formed under physiologically relevant conditions, and its production is solely witnessed in RBCs in vivo subsequent to the administration of oxidizing pharmaceuticals (like phenacetin) together with sulfur-containing compounds, like hydroxylamine sulfate, sulfur dioxide, and others [11].

Notably, methemoglobin forms a reversible metHb-SH^−^ complex with sulfide at more physiologically relevant concentrations, a finding first documented by Kellin in 1933 [68] and later reproduced by others [69,70]. The metHb-SH^−^ adduct was shown to slowly decompose to yield inorganic polysulfides, HS_2_O_3_^−^, and does not react further in the presence of millimolar concentrations of low-molecular-weight thiols, like Cys and GSH [12,69,71].

Recent investigations carried out in intact RBCs have shown that sulfide reacts with metHb to form a metHb-SH^−^ intermediate and produces polysulfide and thiosulfate [12]. Interestingly, the sulfide reactivity with metHb varies in intact cells compared to cell-free solutions. In cell-free hemoglobin solutions, the metHb-SH^−^ complex is fairly stable. Instead, in intact cells, the rapid decomposition of the metHb-SH^−^ complex leads to the formation of deoxyhemoglobin and, in the presence of oxygen, oxyhemoglobin (deoxy/oxyHb). Thus, according to these experiments, treating RBCs with sulfide leads to the reduction of methemoglobin to deoxy/oxyHb. Clearly, there must be cellular mechanisms within RBCs that facilitate metHb-SH^−^ reduction since this occurs in intact cells only and not in hemoglobin solutions.

In vivo, metHb is ubiquitously present in high concentrations within healthy RBC (ranging from 100 to 300 µM), comprising 1–3% of the total hemoglobin concentration. Therefore, metHb is a highly efficacious sulfide scavenger in vivo. As mentioned in the second chapter of this review, the conversion of metHb (which has a low affinity for oxygen) into deoxy/oxyHb (which is the oxygen-binding form) is carried out by a cytochrome C reductase, and it is key for the physiological function of RBCs. The data discussed here show that the same reaction occurs when RBCs are treated with sulfide. Accordingly, sulfide has been shown to reduce heme-containing proteins of mammalian and invertebrate origin [72]. Thus, it is tempting to speculate that in this way, endogenous sulfide may help maintain hemoglobin in an oxygen-binding state. This hypothesis needs to be verified experimentally.

Interestingly, RBCs also express 3-mercaptopyruvate sulfotransferase (3-MST, EC 2.8.1.2) [73], which may catalyze the formation of sulfur species and sulfide inside the cells [69,74,75,76,77,78,79]. 3-MST metabolizes molecules containing sulfane sulfur, i.e., a sulfur atom bound to another sulfur atom (RS-(S)_n_-R′). High 3-MST activity has been measured in RBCs, the liver, kidneys, and adrenal cortex [75,76,77]. Its endogenous substrate is 3-mercaptopyruvate (3-MP), which is generated from l-cysteine and α-ketoglutarate by a cysteine aminotransferase. The main function of 3-MST is to transfer the sulfur atom of 3-MP to acceptors like cyanide, sulfite, and sulfinate, producing soluble compounds for detoxification. This process involves two steps: the formation of MST-bound persulfide and H_2_S. 3-MST also uses HS_2_O_3_^-^ as a substrate to produce sulfite and enzyme-bound sulfane sulfur. Patients with 3-mercapto lactate-cysteine disulfiduria and iron deficiency anemia show higher activity as compared to other patient cohorts and healthy individuals [75,76]. However, the role of 3-MST in erythropoiesis, RBCs physiology, and pathophysiology is unknown.

Besides 3-MST, RBCs contain high concentrations of several other enzymatic systems, like the glutathione system and antioxidant enzymes, which are potentially involved in sulfide/polysulfide metabolism [80,81,82].

There is evidence that RBCs might also participate in the biochemical and biological interaction and “cross-talk” between NO and sulfide [11]. We have shown that NO-induced methemoglobin (metHb) formation can be reversed by sulfide, which reconverts metHb back to deoxy/oxyhemoglobin, effectively restoring the oxygen-carrying capacity [12]. Moreover, we found that sulfide treatment via continuous Na_2_S infusion increased nitrosyl-Hb levels in RBCs in rats, indicating that sulfide boosts the formation of nitrosyl-Hb (considered by some as a vasodilator) [14]. These findings point to an in vivo cross-talk between NO and H_2_S in RBCs, which may occur via the formation of hybrid S/N molecules. The role and effects of this NO/H_2_S cross-talk and its interaction with hemoglobin and antioxidant enzymes on RBC physiology and function remain unknown and warrant further investigation.

## 5. Summary and Perspective

This article discusses how the concept of the *reactive species interactome* pertains to the complex redox biology and physiology of RBCs. At the center of the reactive species interactome in RBC is hemoglobin and its different redox forms, i.e., the reduced form (oxy/deoxyHb) and the oxidized form (metHb). The degradation of metHb (which constitutes 1–3% of all forms) is the main source of the production of superoxide and other oxidants. This is efficiently and potently neutralized by a battery of antioxidant systems. Moreover, oxy/deoxyHb are also involved in NO metabolism, as oxyHb scavenges NO, and deoxyHb reacts with NO (forming HbNO) and produces NO from nitrite. An intracellular source of NO in RBCs is eNOS, which may participate in the formation of NO adducts of hemoglobin, like HbNO. RBCs, therefore, strongly contribute to the systemic regulation of NO metabolism and bioactivity. In addition, metHb is involved in sulfide scavenging and metabolism. Sulfide forms a stable adduct with metHb, which is then reduced—in intact cells only—into deoxy/oxyHb. 3-MST is also a potential source of sulfide/persulfide in RBCs, but its role is still elusive. The exact role that the reactive species interactome plays in the regulation of RBC physiology and pathophysiology remains an open question that needs further investigation.

## Data Availability

No new data were created or analyzed in this study. Data sharing is not applicable to this article.
